# Age-Related Comparative Study of In-Hospital Mortality, Functional Outcome, and Recurrence in a Large Cohort of Patients Surgically Treated for Chronic Subdural Hematoma

**DOI:** 10.3390/jcm14217856

**Published:** 2025-11-05

**Authors:** Schahin Salmanian, Jan Rodemerk, Sali Al-Rubaiey, Madiha Ahmadzai, Elias Timner, Lisa Schock, Thiemo Florin Dinger, Oliver Gembruch, Ramazan Jabbarli, Philipp Dammann, Ulrich Sure, Mehdi Chihi

**Affiliations:** 1 Department of Neurosurgery and Spine Surgery, University Hospital Essen, Hufelandstrasse 55, 45147 Essen, Germany; ssalmanian@usf.edu (S.S.);; 2 Morsani College of Medicine, University of South Florida, Tampa, FL 33602, USA; 3 Department of Anesthesiology and Intensive Care Medicine, University Hospital Essen, 45147 Essen, Germany; 4 Faculty of Medicine, University of Duisburg-Essen, 45147 Essen, Germany; 5 Center for Translational Neuro- and Behavioral Sciences, University Hospital Essen, 45147 Essen, Germany

**Keywords:** chronic subdural hematoma, elderly, outcome, mortality

## Abstract

**Background/Objectives:** Chronic subdural hematoma (CSDH) predominantly affects the elderly population. To optimize care and quality in this demographic, tailored, age-specific counseling and therapeutic decision-making are imperative. Accordingly, this study aimed to identify risk factors for in-hospital mortality and functional outcome at discharge following surgery using an age-stratified approach. **Methods:** We conducted a retrospective analysis of symptomatic CSDH patients who underwent surgery at our institution between June 2012 and December 2023. Subjects were categorized into three age cohorts: younger adults (18–64 years), older adults (65–79 years), and the oldest old (≥80 years). Clinical and neurological statuses at admission and discharge were evaluated using the Glasgow Coma Scale (GCS) and modified Rankin Scale (mRS), with mRS scores > 3 indicating poor functional outcomes. **Results:** Among 879 CSDH patients (mean age 75 ± 11.9 years), the sex ratio shifted progressively from a male predominance in younger adults (1:3.2) to a more balanced ratio in the oldest old (1:1.7). In the multivariate analysis, poor admission mRS and GCS score ≤ 7 predicted in-hospital mortality for older adults, while atrial fibrillation and postoperative pneumonia were significant in the oldest old. Poor admission mRS and multimorbidity consistently forecast unfavorable outcomes alongside other predictors, such as preoperative altered state of consciousness, epilepsy, dementia, unilateral CSDH, postoperative seizure, bleeding, and pneumonia varying by age cohort. Recurrence-free intervals were significantly extended with increasing age. **Conclusions:** This large-scale, age-stratified analysis delineates critical predictors of in-hospital mortality and unfavorable functional outcomes in surgically treated CSDH patients. These findings offer valuable guidance for neurosurgeons in preoperative risk assessment and inform age-specific counseling strategies to better communicate prognosis and tailor treatment plans.

## 1. Introduction

Chronic subdural hematoma (CSDH) is one of the most common neurosurgical conditions, with a markedly increased incidence in aging populations due to demographic shifts and greater longevity worldwide [1,2]. Epidemiological data indicate an annual incidence of approximately 58.1 per 100,000 in individuals over 65 years of age, compared to only 3.4 per 100,000 in those under 65 [2,3]. The disease typically arises from minor head traumas, leading to bridging vein rupture, followed by progressive hematoma expansion driven by fragile neo-membrane vasculature and inflammatory cascades [4,5]. While CSDH is traditionally associated with elderly patients, often presenting with neurological deficits such as limb weakness, gait disturbance, or cognitive impairment, it can also occur in younger adults, in whom symptoms more frequently reflect raised intracranial pressure, including headache and vomiting [6,7,8,9,10].

The clinical presentation, comorbidity profile, and prognosis of CSDH vary substantially with age. Younger patients often have identifiable predisposing factors, such as arachnoid cysts, ventriculoperitoneal shunts, or coagulation disorders, whereas elderly patients more commonly exhibit multimorbidity, brain atrophy, and antithrombotic therapy use [2,4,11,12,13]. Comparative studies have shown that while recurrence rates after surgery are not consistently higher in older cohorts, postoperative complication rates and in-hospital mortality increase with advancing age [2,8]. Moreover, functional outcomes tend to be more favorable in younger patients [2,14,15]. This underscores the importance of incorporating age-stratified risk assessment into both prognostication and perioperative management.

Given these age-dependent variations in presentation, comorbidity burden, and postoperative trajectories, a more granular, age-stratified evaluation of CSDH patients is warranted to optimize prognostication, guide perioperative management, and inform shared decision-making. Although several studies have compared surgical outcomes between younger and older adults, most have employed a dichotomous age classification or utilized broad age groups, potentially obscuring clinically relevant differences within the elderly population [2,4,8,10,16,17]. By adopting a three-tier age stratification, our study aims to delineate both common and age-specific predictors of in-hospital mortality and functional outcome at discharge in a large, contemporary surgical CSDH cohort. This approach allows for the identification of distinct risk profiles across the adult lifespan, thereby enabling more tailored perioperative strategies and evidence-based counseling for patients and their families.

## 2. Methods and Materials

### 2.1. Study Population

**Figure 1:**
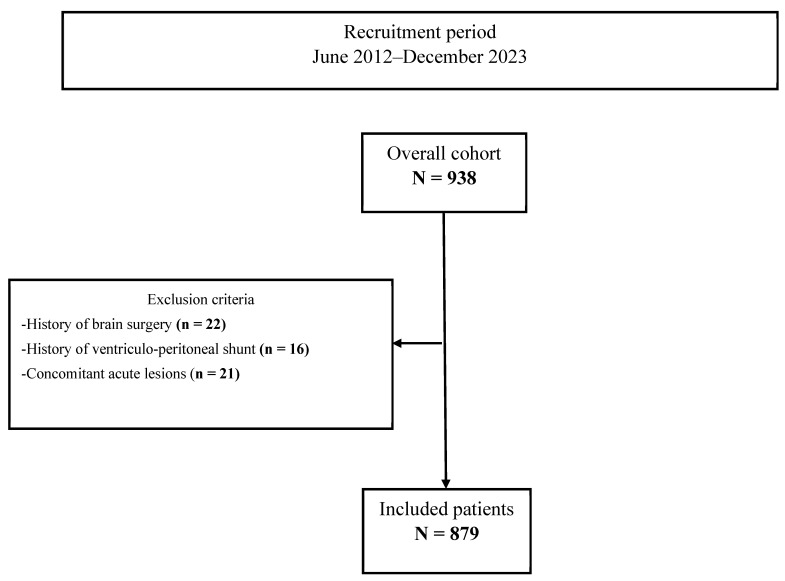
Study flowchart.

Between June 2012 and December 2023, 938 consecutive patients aged ≥18 years with symptomatic CSDH admitted at our institution were evaluated for the eligibility for this study. Patients with a history of brain surgery (intracranial tumor, acute subdural hematoma, aneurysm clipping, and CSDH), ventriculoperitoneal shunt, as well as patients with concomitant acute lesions (intracranial hemorrhage, traumatic brain injury) were excluded. The study flowchart shows the selection process and the main exclusion criteria in detail (Figure 1).

This retrospective study was performed in accordance with the ethical standards of the institutional research committee and the Code of Ethics of the World Medical Association (Declaration of Helsinki). The Institutional Review Board approved the study protocol (Medical Faculty, University of Duisburg-Essen, Registration number: 22-10763-BO). All supporting data are available within this article. Anonymized data will be shared upon reasonable request from any qualified investigator.

### 2.2. Definition of Age-Related Groups in CSDH Patients

In accordance with contemporary clinical research practices and epidemiological literature, and consistent with German and international standards in gerontology and public health [18,19,20,21], patients were stratified into three age groups: younger adults (18–64 years), older adults (65–79 years), and the oldest old (≥80 years).

### 2.3. Patient Data Collection

The patients’ medical records were retrospectively assessed to examine the following data points: age, sex, Charlson comorbidity Index (CCI), American Society of Anesthesiologists Physical Status Classification System (ASA status), traumatic event, as well as comorbidities such as epilepsy, dementia, arterial hypertension, diabetes mellitus, chronic kidney disease (CKD), atrial fibrillation (AF), chronic heart disease (CHD), cerebral vascular accident or transient ischemic accident (CVA/TIA), prior antiplatelet and/or anticoagulation therapy (AAT)–including acetylsalicylic acid (aspirin), clopidogrel, vitamin K antagonists (VKA), direct oral anticoagulants (DOAC), heparin, dual or triple AAT–seizure 24 h prior to admission, neurological condition, and functional status on admission using the GCS score and the modified Rankin Scale (mRS), respectively. Furthermore, the variable “altered state of consciousness” was assessed and defined as changes in awareness or perception that deviate qualitatively from a person’s usual, alert, and oriented condition. Radiographic variables, such as midline shift (MLS), hematoma thickness, presence of septations, and hematoma density, were assessed from the preoperative CT-scans. Along with the patients’ period of hospitalization, operative technique (one burr hole vs. minicraniotomy–unilateral CSDH–vs. two burr holes vs. minicraniotomy and one burr hole–bilateral CSDH), neurological condition on discharge, in-hospital mortality, and postoperative complications, such as postoperative bleeding requiring reoperation, postoperative seizure, recurrence, myocardial infarction (MI), pulmonary embolism (PE), and postoperative stroke were assessed. Recurrence was defined as an episode of radiological hematoma progression, with or without development of symptoms, that required reoperation. Functional status of the patients was also documented at discharge. Patients with a postoperative mRS score ≤ 3 were able to walk without assistance and although moderately disabled could be discharged home. Patients with an mRS score equal to 4 or 5 were transferred to rehabilitation. Therefore, an mRS score > 3 was considered to have a poor outcome. Prior to statistical analysis, the data were thoroughly reviewed and validated by the senior author (M.C.).

### 2.4. Routine Clinical Care and Treatment Protocol of CSDH

In all patients, hematoma evacuation was performed under general anesthesia using either a burr hole or a minicraniotomy with placement of a subdural drain whenever possible. Prior to surgery, a single dose of antibiotics was administrated prophylactically. Postoperatively, patients spent 48 h in the intermediate or intensive care unit and remained on bed rest. On the second postoperative day, a CT-scan was performed and the subdural drain was removed. Patients were usually discharged around day 7 after surgery.

### 2.5. Perioperative Management of AAT

AAT was discontinued prior to surgery (aspirin and clopidogrel 5–7 days, DOAC 48 h, and VKA until the Quick value exceeded 70%, i.e., INR < 1.3). AAT was reinitiated three weeks after surgery if the hematoma had completely resolved. In cases of neurological deterioration during the discontinuation period, Desmopressin or PPSB was administered to reverse the effects of aspirin and VKA, respectively, in order to normalize coagulation status and enable a safe emergency operation. Patients in whom AAT could not be discontinued (*n* = 18) either underwent surgery while continuing AAT (*n* = 13) or had at least one of the two antithrombotic drugs temporarily stopped. Patients, for whom AAT discontinuation was advised to be as brief as possible (*n* = 9), were operated on after a temporary cessation of AAT and were subsequently bridged with heparin during the first postoperative week until oral anticoagulation could be safely resumed.

### 2.6. Assessment of Radiographic Variables

Radiographic parameters were assessed from preoperative axial CT-scans. Hematoma thickness was measured as the maximum perpendicular thickness from the inner skull table to the inner margin of the hematoma. In case of bilateral hematomas, the sum of both maximum thicknesses was considered to represent total hematoma burden. MLS was measured at the foramen of Monro as the perpendicular distance from the ideal midline–connecting the most anterior and posterior visible points of the falx cerebri–to the septum pellucidum. The density of the hematoma was divided in hypodense, isodense, hyperdense, or mixed density. Septations within the hematoma were also assessed.

### 2.7. Statistical Analyses

All analyses were performed using SPSS version 29.0.2.0 (IBM, Chicago, IL, USA). Categorical data are presented as frequencies and percentages. Normally distributed continuous variables are expressed as the mean and standard deviation (SD). Non-normally distributed continuous variables are expressed as the median and interquartile range (IQR). The Mann–Whitney U test was used to assess statistical associations between the study endpoints (in-hospital mortality, functional outcome at discharge) and other continuous variables. Associations between study endpoints and other dichotomous variables were determined using the Chi-square test and Fisher’s exact test (when the expected cell frequency was <5). The factors in the univariate analysis, which were associated with the study endpoints with a *p*-value < 0.1, were implemented in the multivariate analysis. Binomial logistic regression (forward likelihood ratio method) was used to determine the predictors of the study endpoints, and the adjusted odds ratio (aOR) and 95% confidence intervals (95% CI) were calculated. Prediction models based on the results of the multivariate analysis were computed. *p*-values < 0.05 in two-sided testing were considered statistically significant. Receiver operating characteristic (ROC) curve analyses were conducted to determine the accuracy of the prediction models. Events per variable (EPV) between five and nine were considered to yield acceptable bias and coverage under well-behaved conditions [22]. For EPV lower than five, internal validation and bias correction of the logistic regression models was conducted by bootstrapping with 1000 resamples. Bias-corrected and accelerated (BCa) 95% CI were computed for all regression coefficients. Subsequently, using R software (version 4.5.1, R Foundation for Statistical Computing, Vienna, Austria), ROC curve analysis was conducted to evaluate the discriminatory ability of the model. Optimism-corrected AUC values and its 95% CIs were calculated using bootstrap resampling (1000 replicates) to internally validate the predictive performance.

### 2.8. Use of GenAI in Writing and Editing

AI-assisted copy-editing using Perplexity AI (GPT-4 architecture) was utilized to improve the readability, grammar, and style of the manuscript. This process involved refining human-generated text without altering the scientific content. All final text revisions were reviewed and approved by the authors to ensure accuracy and integrity.

## 3. Results

### 3.1. Baseline Characteristics

Accordingly, 879 patients (mean age: 75 ± 11.9 years, female:male = 1:2.1, CCI: 4 (3), ASA status: 3 (0)) met the inclusion criteria. There were no statistically significant differences in the operative technique (*p* = 0.929) and CSDH location (*p* = 0.827) between the three age-related groups. However, statistically significant differences were observed in sex ratio (*p* = 0.001), CCI (*p* < 0.001), ASA status (*p* < 0.001), recent head trauma, several comorbidities (*p* < 0.001), as well as prior AAT use (*p* < 0.001), preoperative symptoms and neurological deficits (*p* < 0.05), postoperative pneumonia (*p* = 0.023), total hospital stay (*p* = 0.008), mRS at admission and discharge (*p* = 0.0005), and in-hospital mortality (*p* = 0.02). The sex ratio evolved from a strong predominance of males (female:male 1:3.2), gradually shifting to a less pronounced male predominance (1:2.4), and approaching a more balanced distribution (1:1.7). CCI and ASA status were highest in the oldest old. Comorbidities, such as dementia, arterial hypertension, diabetes mellitus, CKD, AF, CHD, and CVA/TIA, increased in frequency with age. In contrast, preoperative symptoms, such as nausea, headache, and seizure within 24 h prior to admission, were inversely related to age. Neurological deficits—including aphasia, dysarthria, hemiparesis—altered state of consciousness, cognitive decline, gait disturbances at admission, as well as functional statuses at admission and discharge, and in-hospital mortality, were all positively associated with advancing age. Preoperative CT-based severity criteria, such as hematoma thickness, midline shift greater than 10 mm, and hematoma density (especially mixed density), were significantly more frequent with increasing age (*p* < 0.001, *p* = 0.002, and *p* = 0.005, respectively). Postoperative pneumonia was also more frequent in the oldest old. Total length of hospital stay increased proportionally with age. Table 1 shows in detail all these findings.

### 3.2. Characteristics and Predictors of In-Hospital Mortality

In-hospital mortality was 3.6% in the general cohort and substantially increased from 1.4% in younger adults, to 2.9% in older adults, reaching up to 5.2% in the oldest old (*p* = 0.020). In the univariate analysis, in-hospital mortality among younger adults was significantly associated with age (*p* = 0.031) and CCI (*p* = 0.002). Postoperative bleeding requiring reoperation (*p* = 0.080) demonstrated a non-significant trend. Multivariate analysis identified no independent predictors of in-hospital mortality in this cohort (Table 2A).

**Figure 2:**
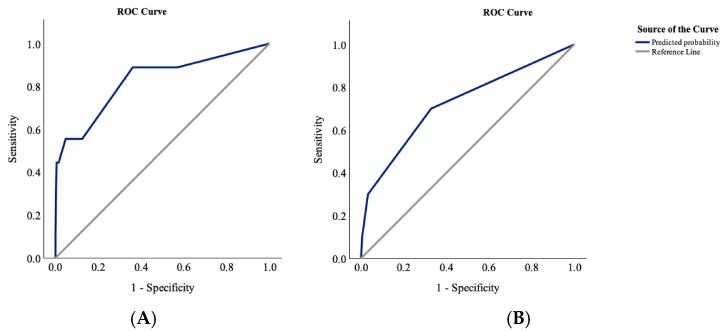
ROC curve analyses of in-hospital mortality showing an AUC of 0.83 (95% CI 0.66–0.99) in older adults (**A**) and an AUC of 0.72 (95% CI 0.59–0.85) in the oldest old (**B**).

In the older adult group, univariate analysis revealed significant associations between in-hospital mortality and CCI (*p* = 0.017), operative technique (*p* = 0.005), mRS at admission (*p* = 0.016), preoperative altered state of consciousness (*p* = 0.048), preoperative GCS score ≤ 7 (*p* = 0.009), and third recurrence (*p* = 0.029). ASA status (*p* = 0.078), preoperative hemiplegia (*p* = 0.084), and postoperative bleeding requiring reoperation (*p* = 0.059) exhibited non-significant trends. In the multivariate model, mRS at admission (*p* = 0.041) and preoperative GCS score ≤ 7 (*p* = 0.040) were identified as independent predictors of in-hospital mortality, with an EPV of 5 (Table 2). The model’s discrimination ability, was indicated by an AUC of 0.83 (95% CI 0.66–0.99) in the ROC curve analysis (Figure 2A).

Among the oldest old patients, in-hospital mortality was significantly associated with ASA status (*p* = 0.043), epilepsy (*p* = 0.037), postoperative bleeding requiring reoperation (*p* < 0.001), and second recurrence (*p* = 0.014). Non-significant trends were observed for diabetes mellitus (*p* = 0.070), AF (*p* = 0.081), prior AAT use (*p* = 0.057), preoperative aphasia (*p* = 0.092), postoperative pneumonia (*p* = 0.054), and third recurrence (*p* = 0.052). In multivariate analysis, AF (*p* = 0.031) and postoperative pneumonia (*p* < 0.001) emerged as independent predictors of in-hospital mortality in this group, with an EPV of 10 (Table 2). The model’s discriminative capacity was determined by an ROC curve analysis and was, according to Hosmer et al. [23], acceptable with an AUC of 0.72 (95% CI 0.59–0.85) (Figure 2B).

### 3.3. Characteristics and Predictors of Functional Outcome at Discharge

Unfavorable functional outcome at discharge consistently increased from 14.3% in younger adults, to 28.2% in older adults, reaching up to 53.8% in the oldest old (*p* = 0.0005).

**Figure 3:**
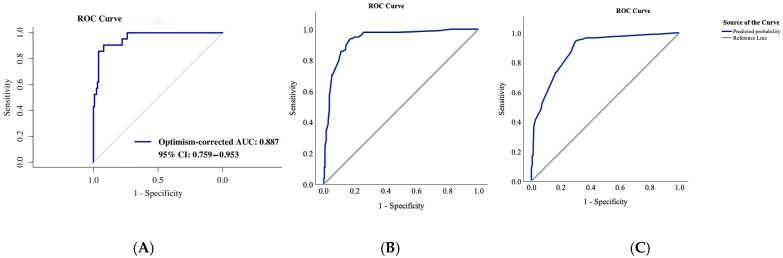
ROC curve analyses of functional outcome at discharge showing an optimism-corrected AUC of 0.89 (95% CI: 0.76–0.95) in younger adults (**A**), an AUC of 0.94 (95% CI: 0.91–0.96) in the older adults (**B**), and an AUC of 0.88 (95% CI: 0.85–0.92) in the oldest old (**C**).

In the younger adult cohort, univariate analysis demonstrated that poorer functional outcomes at discharge were significantly associated with several factors: age (*p* = 0.038), sex (*p* = 0.049), ASA status (*p* < 0.001), admission mRS score (*p* < 0.001), presence of preoperative headache (*p* = 0.001), altered state of consciousness (*p* < 0.001), aphasia (*p* = 0.008), hemiparesis (*p* = 0.030), and postoperative seizures (*p* = 0.010). Variables such as CCI (*p* = 0.075), epilepsy (*p* = 0.052), behavioral changes prior to admission (*p* = 0.071), and preoperative neurological deficits (*p* = 0.058) approached but did not reach statistical significance. In multivariate analysis, significant independent predictors of poor functional outcome at discharge included female sex (*p* = 0.040), elevated ASA status (*p* = 0.010), epilepsy (*p* = 0.029), poor mRS at admission (*p* < 0.001), and postoperative seizure (*p* = 0.005) (Table 3A,B). Due to low EPV logistic regression model (EPV = 4.2), an internal validation was conducted using bootstrapping. This analysis showed that only elevated ASA status (*p* = 0.002, Bca 95% CI: 1.1–414.7), poor mRS at admission (*p* = 0.001, Bca 95% CI: 4–971.5), and postoperative seizure (*p* = 0.002, Bca 95% CI: 0.5–614.1) were independent predictors of poor functional outcome at discharge (Table 4). The predictive performance of the multivariate model was assessed using an ROC curve analysis in R software and showed an optimism-corrected AUC of 0.89 (95% CI: 0.76–0.95) (Figure 3A).

In older adults, univariate analysis found functional outcome at discharge to be significantly impacted by age (*p* = 0.012), CCI (*p* < 0.001), ASA status (*p* < 0.001), epilepsy (*p* = 0.009), dementia (*p* < 0.001), diabetes mellitus (*p* = 0.001), CKD (*p* = 0.01), AF (*p* = 0.038), CVA/TIA (*p* = 0.009), CSDH location (*p* = 0.043), year of surgery (*p* = 0.045), prior AAT use (*p* = 0.031), perioperative AAT stop and heparin bridging (*p* = 0.024), and mRS at admission (*p* < 0.001). Other associated variables included preoperative headache (*p* < 0.001), change in behavior (*p* = 0.049), cognitive deterioration (*p* < 0.001), seizure 24 h prior to admission (*p* = 0.035), neurological deficits (*p* = 0.005), altered state of consciousness (*p* < 0.001), preoperative GCS score ≤ 7 (*p* < 0.001), hemiparesis (*p* = 0.008), hemiplegia (*p* = 0.022), mixed hematoma density on CT-scan (*p* = 0.018), postoperative seizures (*p* < 0.001), postoperative bleeding requiring reoperation (*p* = 0.026), and postoperative pneumonia (*p* < 0.001). Trends toward significance were noted with dizziness (*p* = 0.091) and dysarthria (*p* = 0.073) (Table 3A). Multivariate analysis identified several independent predictors of poor discharge outcomes: high CCI (*p* = 0.036), dementia (*p* = 0.016), unilateral CSDH (*p* = 0.016), poor mRS at admission (*p* < 0.001), and postoperative pneumonia (*p* = 0.013), with an EPV of 19.6 (Table 3B). The model’s discriminative accuracy, evaluated via ROC analysis, was outstanding according to Hosmer et al. [23]. with an AUC of 0.94 (95% CI: 0.91–0.96) (Figure 3B).

For the oldest old subgroup, univariate associations revealed that impaired functional outcomes at discharge were significantly related to age (*p* = 0.004), CCI (*p* < 0.001), ASA status (*p* < 0.001), recent head trauma (*p* = 0.038), epilepsy (*p* = 0.015), dementia (*p* < 0.001), diabetes mellitus (*p* = 0.029), poor mRS at admission (*p* < 0.001), and presenting preoperative symptoms such as headache (*p* = 0.034), dizziness (*p* = 0.018), cognitive deterioration (*p* = 0.003), and altered state of consciousness (*p* < 0.001). Additional relevant factors included preoperative aphasia (*p* = 0.002), postoperative seizure (*p* = 0.006), postoperative bleeding requiring reoperation (*p* = 0.019), and postoperative pneumonia (*p* < 0.001). Arterial hypertension (*p* = 0.081), CKD (*p* = 0.054), year of surgery (*p* = 0.089), prior AAT use (*p* = 0.070), change in behavior (*p* = 0.089), preoperative GCS score ≤ 7 (*p* = 0.064), hematoma thickness (*p* = 0.057), and mixed hematoma density on CT-scan (*p* = 0.053) showed non-significant trends (Table 3A). Multivariate analysis identified several independent predictors of poor outcome at discharge: elevated ASA status (*p* = 0.014), epilepsy (*p* = 0.023), dementia (*p* = 0.003), poor mRS at admission (*p* < 0.001), altered state of consciousness (*p* = 0.035), and postoperative bleeding requiring reoperation (*p* = 0.007), with an EPV of 34.5 (Table 3B). The model’s discriminative strength, as assessed via ROC curve, was excellent according to Hosmer et al. [23]. with an AUC of 0.88 (95% CI: 0.85–0.92) (Figure 3C).

**Figure 4:**
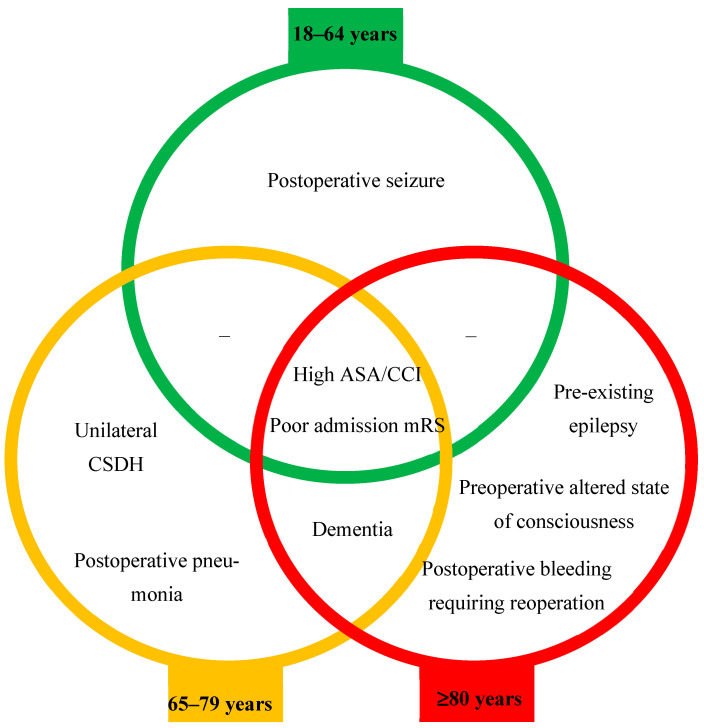
Age-stratified risk assessment of poor functional outcome at discharge.

Figure 4 summarizes all these findings.

### 3.4. Characteristics of Recurrence

**Figure 5:**
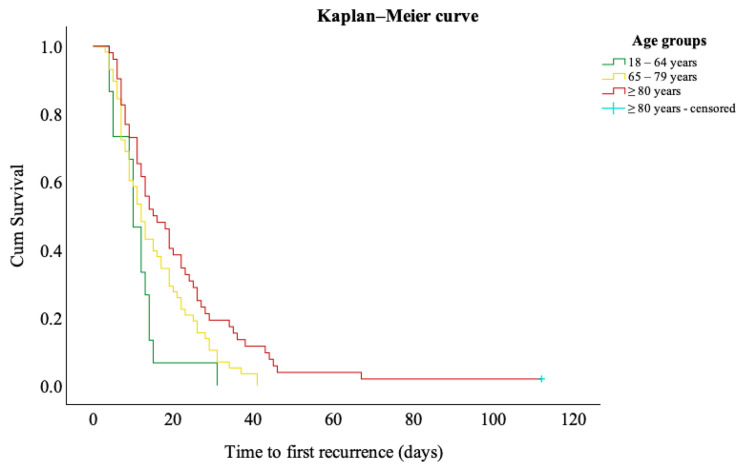
Kaplan–Meier curves for time to first recurrence stratified by age groups.

Interestingly, there was no statistically significant difference in recurrence rates between age groups (*p* = 0.932). However, time to first recurrence increased significantly with age (*p* = 0.001). The Kaplan–Meier survival analysis evaluated time to first recurrence across age groups. Censoring was defined as either death or discharge without recurrence. Mean time to first recurrence with 95% CI was estimated and reported in Table 1. Recurrence-free survival curves for each age group were plotted (Figure 5), and differences were assessed by log-rank test (*p* = 0.006).

## 4. Discussion

Our retrospective study using an age-stratified approach showed that the sex ratio of CSDH patients shifted from a strong male predominance in the group of younger adults toward a more balanced distribution in the oldest old group. Additionally, this approach allowed stratification of age-category independent risk factors for in-hospital mortality and functional outcome at discharge. For in-hospital mortality, poor neurological status at admission—defined by a preoperative GCS score ≤ 7—and poor functional status at admission were key predictors in the older adult group, while AF and postoperative pneumonia were predictors in the oldest old group. Regarding poor functional outcome at discharge, independent predictors, such as poor functional status at admission and multimorbidity, were consistent across all three groups. Dementia was a relevant predictor in the older adult and oldest old groups. Postoperative seizures were significant only in the younger adult group; unilateral CSDH and postoperative pneumonia were relevant in the older adult group; and pre-existing epilepsy, preoperative altered state of consciousness, and postoperative bleeding requiring reoperation were predictors only in the oldest old group.

The evolution of the sex ratio in CSDH is notable for its gradual transition from strong male predominance in the young population toward a more equal distribution in the elderly. This trend is best observed by comparing age-related sex ratios, as demonstrated in our study. Early research in the 1970s reported that males significantly outnumbered females, with female:male ratios near 1:10 [10,24]. Kanat et al. proposed that the higher incidence of CSDH in males may be explained by greater exposure to head injuries among men and the protective role of estrogen on the blood vessel capillaries in women [25]. Over the following decades, this gap narrowed; by the 1980s, the ratio approached 1:3.5–1:4 [10,24], reflecting a rising proportion of elderly women being diagnosed [10], and a shift in the peak age of onset from 50 years in the 1970s to 70 years in 2010 [24]. Recent research documents this trend continuing, with contemporary studies noting a ratio of 1:2.4 [26], which aligns with the sex ratio of the general cohort in our study (1:2.1). This shift is attributed to factors such as increased female longevity and loss of hormonal protection in the postmenopausal period [27]. Although a male predominance persists, demographic changes including aging populations and female health advancements might drive a measurable rise in female CSDH cases, leading to a more equal sex distribution over time. Furthermore, aging populations led to an increase in in-hospital mortality rates and greater disparities in functional outcome at discharge.

In our study, the in-hospital mortality rate was 3.6%, consistent with recent large-scale epidemiological research on CSDH, reporting rates of 3% and 4.2% [1,28]. Furthermore, our findings reveal that in-hospital mortality predictive factors shifted between cohorts. In older adults, it was independently associated with poor admission neurological status and poor functional status at admission, which align with recent research findings [28,29,30,31]. In the oldest old, AF and postoperative pneumonia were the dominant predictors in our study. These factors are in line with recent study findings [28,32] and highlight the vulnerability of this cohort to systemic complications, underlining the importance of early mobilization, pulmonary hygiene [33,34,35], and overall thoughtful evaluation of risks and benefits of treatment modalities for cardiac diseases in this age group [10,36]. In younger adults, in-hospital mortality occurred in only two patients (1.4%), both of whom had a high CCI, indicating significant morbidity.

Regarding functional outcome at discharge, across all age groups in our study, poor functional status at admission and multimorbidity emerged as the most consistent predictors of unfavorable outcomes. These findings confirm that baseline neurological condition and overall systemic disease burden remain the strongest determinants of postoperative trajectory, irrespective of chronological age. The robustness of these associations aligns with the previous literature highlighting mRS as a powerful prognostic marker in CSDH [7,14,37]. Given their modifiable nature is limited, early identification of these factors should guide discussions with patients and families, set realistic expectations, and prompt aggressive optimization of comorbidities before surgery whenever possible.

While poor functional status at admission and multimorbidity were universal predictors of functional outcome at discharge, several age-specific factors emerged. In younger adults, postoperative seizures significantly impaired postoperative functional recovery with an incidence of 7.5%. A recent large cohort study corroborated our results, reporting a lower incidence rate of postoperative seizures (4.2%), though associated with significantly poorer clinical outcomes [38]. While the global prophylactic use of antiepileptic drugs in these patients has shown mixed evidence, individual case-based decision-making may be warranted in a specific set of patients [39,40]. In line with prior mixed-age series linking postoperative pulmonary complications to increased frailty and impaired recovery [41,42,43], our study identified postoperative pneumonia as an independent determinant of poor functional outcome among older adults, suggesting that systemic vulnerability amplifies the neurological burden in this population. Evidence comparing functional outcomes between unilateral and bilateral hematomas remains limited, often based on small, heterogeneous cohorts. Nevertheless, several reports have suggested less favorable outcomes in patients with bilateral CSDH [43,44,45]. In our large and tiered age-related study, we further clarified this issue by noting that unilateral hematomas independently predicted poorer functional outcomes only in older adults. In the oldest old, dementia, altered state of consciousness, pre-existing epilepsy, and postoperative bleeding requiring reoperation were identified as key determinants of outcome. Dementia likely reduces cognitive reserve, thereby impeding rehabilitation potential [5,46,47,48]. Previous studies have found no direct association between postoperative bleeding requiring reoperation and age [49,50], instead linking it to comorbidities such as arterial hypertension and CKD [13], both of which were significantly more prevalent in our study population, especially in the oldest old group. Furthermore, postoperative bleeding was frequently associated with neurological deterioration and disproportionately affected this elderly group, likely due to their increased neurological vulnerability and frailty, which explains its pronounced negative impact on short-term functional outcomes. Pre-existing epilepsy in CSDH patients is relatively uncommon—our study observed an incidence of 3.9% in the oldest old—but remains understudied. Nevertheless, it may increase the risk of postoperative seizures, thereby elevating the likelihood of poor functional outcomes. Our findings identified pre-existing epilepsy as an independent predictor of adverse functional recovery in this population.

Regarding recurrence, several meta-analyses have debated the role of aging, with some supporting an increased risk of recurrence in the elderly [51], while others found no significant association [6]. Our findings demonstrated that age does not affect recurrence risk; however, it had a significant impact on the time to first recurrence. To date, data on age-associated recurrence-free intervals remain scarce. Our findings demonstrated a statistically significant prolongation of the first recurrence-free interval with increasing age.

Our findings argue for a tiered, age-specific risk stratification model in CSDH surgery. In younger adults, optimizing seizure control could improve recovery. In older adults, the importance of timely surgical evacuation of unilateral CSDH and aggressive prevention of postoperative pneumonia are key. In the oldest old, meticulous surgical planning, strict intraoperative hemostasis to minimize the risk of postoperative bleeding requiring reoperation, and implementation of multidisciplinary strategies targeting comorbidities (such as pre-existing epilepsy and dementia), alongside tailored rehabilitation for cognitive impairment are paramount. Importantly, the absence of significant differences in recurrence rates between age groups suggests that age alone should not dictate surgical candidacy; rather, comorbidity and functional reserve should guide decision-making. Moreover, clinical and radiological vigilance regarding prolonged recurrence-free intervals in older adults and the oldest old are crucial.

## 5. Limitations

This is a single-center retrospective study, which may limit generalizability of its findings, as institutional protocols and surgical expertise can significantly influence outcomes. Conducted at a tertiary care center in a large Western European city, referral patterns and baseline patient characteristics may differ from those at other institutions. While the large cohort size enhances statistical power, it introduces temporal heterogeneity, since evolving standards of care, including changes in operative techniques and reoperation strategies with the recent adoption of middle meningeal artery embolization, may impact outcomes and thresholds for reoperations over the study period. To mitigate this, the variable “year of surgery” was incorporated into the statistical analyses.

## 6. Conclusions

In surgically treated CSDH patients, age-stratified analysis reveals that poor baseline function and multimorbidity are universal predictors of unfavorable outcomes, while other risk factors vary with age. Older adults are most vulnerable to mortality from poor neurological and functional statuses at admission, whereas the oldest old are primarily threatened by pulmonary complications and cardiovascular history. Functional recovery is shaped by both shared and age-specific factors, including postoperative seizures in the younger adults; unilateral hematomas, dementia, and postoperative pneumonia in the older adults; pre-existing epilepsy, preoperative altered state of consciousness, postoperative bleeding requiring reoperation, and dementia in the oldest old. Moreover, recurrence-free intervals are prolonged with increasing age. These findings underscore the importance of individualized perioperative strategies that integrate age-specific risk profiles into surgical decision-making, counseling, and postoperative care planning.

## Figures and Tables

**Table 1:** Relevant differences in baseline characteristics between age groups.

Variables	Age Groups (Years)			Total	*p*-Value
	18–64 (*n* = 147)	65–79 (*n* = 347)	≥80 (*n* = 385)		
Age (mean ± SD)	54.5 ± 9.6	73.4 ± 4.2	84.8 ± 3.6	75.2 ± 11.9	**--**
Sex (ratio female:male)	1:3.2	1:2.4	1:1.7	1:2.1	0.001
Charlson comorbidity index (median (IQR))	2 (1)	4 (2)	5 (2)	4 (3)	<0.001
ASA status (median (IQR))	3 (1)	3 (1)	3 (0)	3 (0)	<0.001
Recent head trauma	91/61.9	254/73.2	322/83.6	667/75.9	<0.001
Epilepsy	11/7.5	15/4.3	15/3.9	41/4.7	0.150
Dementia	2/1.4	41/11.8	94/24.4	137/15.6	<0.001
Arterial hypertension	67/45.6	251/72.3	285/74	603/68.6	<0.001
Diabetes mellitus	16/10.9	82/23.6	103/26.8	201/22.9	<0.001
Chronic kidney disease	2/1.4	28/8.1	63/16.4	93/10.6	<0.001
Atrial fibrillation	4/2.7	70/20.2	120/31.2	194/22.1	<0.001
Chronic heart disease	12/8.2	66/19	96/24.9	174/19.8	<0.001
Cerebral vascular/transient ischemic accident	11/7.5	47/13.5	69/17.9	127/14.4	0.002
Operative technique (one burr hole)	100/68	232/66.9	263/68.3	595/67.7	0.827
CSDH location (bilateral)	28/19	76/21.9	79/20.5	183/20.8	0.937
Prior AAT use	37/25.2	183/52.7	250/64.9	470/53.5	<0.001
-Aspirin	22/15	81/23.3	121/31.4	224/25.5	-
-Clopidogrel	0/0	3/0.9	9/2.3	12/1.4	-
-VKA	4/2.7	61/17.6	58/15.1	123/14	-
-DOAC	6/4.1	18/5.2	34/8.9	58/6.6	-
-Dual or triple therapy	4/2.7	20/5.8	27/7	51/5.8	-
-Heparin	1/0.7	0/0	1/0.3	2/0.2	-
Perioperative AAT short stop and heparin bridging	0/0	5/1.4	4/1	9/1	0.451
No perioperative stop of AAT	3/2	8/2.3	7/1.8	18/2	0.949
At admission: mRS > 3	36/24.5	131/37.8	247/64.2	414/47.1	0.0005
Preoperative symptoms and signs					
-Nausea	15/10.2	21/6.1	13/3.4	49/5.6	0.003
-Headache	91/61.9	123/35.4	97/25.2	311/35.4	<0.001
-Dizziness	25/17	51/14.7	46/11.9	122/13.9	0.108
-Change in behavior	17/11.6	55/15.9	59/15.3	131/14.9	0.465
-Cognitive deterioration	39/26.5	114/32.9	148/38.4	301/34.2	0.008
-Gait disorder	55/37.4	161/46.4	221/57.4	437/49.7	<0.001
-Seizure < 24 h	13/8.8	25/7.2	14/3.6	52/5.9	0.009
-Neurological deficits	74/50.3	213/61.4	259/67.3	546/62.1	<0.001
-Altered state of consciousness	48/32.7	157/45.2	205/53.2	410/46.6	<0.001
-GCS score ≤ 7	2/1.4	6/1.7	5/1.3	13/1.5	0.784
-Aphasia	29/19.7	112/32.3	138/35.8	279/31.7	0.002
-Dysarthria	6/4.1	27/7.8	54/14	87/9.9	<0.001
-Hemiparesis	63/42.9	187/53.9	237/61.6	487/55.4	<0.001
-Hemiplegia	1/0.7	3/0.9	6/1.6	10/1.1	0.302
-Sensitive deficit	23/15.6	36/10.4	43/11.2	102/11.6	0.349
Preoperative CT-based severity criteria	136/92.5	307/88.5	833/87.8	781/88.9	
-Hematoma thickness (mean ± SD in mm)	22.9 ± 8.4	24.9 ± 9.2	26.8 ± 12.6	25.4 ± 10.8	<0.001
-Midline shift > 10 mm	50/36.8	89/29	77/22.8	216/24.6	0.002
-Presence of septations	62/45.6	176/57.3	194/57.4	432/49.1	0.072
-Density					
•hypodense	29/21.3	50/16.3	58/17.2	137/15.6	0.005
•isodense	21/15.4	38/12.4	22/6.5	81/9.2	
•hyperdense	9/6.6	21/6.8	16/4.7	46/5.2	
•mixed	77/56.6	198/64.5	242/71.6	517/58.8	
Postoperative complications					
-Seizure	11/7.5	21/6.1	29/7.5	61/6.9	0.717
-Bleeding requiring re-operation	6/4.1	33/9.5	29/7.5	68/7.7	0.575
-Pneumonia	2/1.4	8/2.3	18/4.7	28/3.2	0.023
-Pulmonary embolism	0/0	1/0.3	4/1	5/0.6	0.096
-Myocardial infarction	0/0	0/0	3/0.8	3/0.3	0.067
-Stroke	1/0.7	0/0	2/0.5	3/0.3	0.752
New neurological deficit at discharge	6/4.1	15/4.3	15/3.9	36/4.1	0.841
New motor deficit at discharge	3/2	8/2.3	7/1.8	18/2	0.744
New sensitive deficit at discharge	2/1.4	0/0	1/0.3	3/0.3	0.231
At discharge: mRS > 3	21/14.3	98/28.2	207/53.8	326/37.1	0.0005
Total hospital stay in days	10.4 ± 8.1	11.5 ± 7.3	11.5 ± 6.3	11.3 ± 7	0.008
First Recurrence	15/10.2	58/16.7	51/13.2	24/14.1	0.932
Time to first recurrence in days (mean (95% CI))	11.2 (7.8–14.6)	15.4 (12.8–18)	21.4 (16.4–26.3)	17.4 (14.9–19.9)	0.011
Second recurrence	2/1.4	11/3.2	4/1	17/1.9	0.270
Third recurrence	0/0	1/0.3	1/0.3	2/0.2	0.699
In-hospital mortality	2/1.4	10/2.9	20/5.2	32/3.6	0.020

Abbreviations: AAT: antiplatelet and/or anticoagulation therapy, ASA status: Anesthesiologists Physical Status Classification System, CSDH: chronic subdural hematoma, CI: confidence intervals, CT: computed tomography, DOAC: direct oral anticoagulants, GCS: Glasgow coma scale, IQR: interquartile range, mRS: modified Rankin scale, SD: standard deviation, VKA: vitamin K antagonists.

**Table 2:** (**A**): Predictors of in-hospital mortality in different age groups. (**B**): Predictors of in-hospital mortality in different age groups.

(**A**)									
**Variables**	**Age Groups (Years)**								
	**18–64** **(*n* = 147)**			**65–79** **(*n* = 347)**			**≥80** **(*n* = 385)**		
**Univariate Analysis**									
	**Mean ± SD**		***p*** **-Value**	**Mean ± SD**		***p*** **-Value**	**Mean ± SD**		***p*** **-Value**
Age (mean ± SD)	63.5 ± 0.7		0.031	74.3 ± 4.4		0.457	85.9 ± 4.1		0.185
	**Median (IQR)**		***p*** **-value**	**Median (IQR)**		***p*** **-value**	**Median (IQR)**		***p*** **-value**
Charlson comorbidity index (median (IQR))	8 (0)		0.002	4.5 (7)		0.017	5.5 (3)		0.129
ASA status (median (IQR))	3 (0)		0.403	3 (1)		0.078	3 (0)		0.043
	***n*** **/%**		***p*** **-value**	***n*** **/%**		***p*** **-value**	***n*** **/%**		***p*** **-value**
Sex (female)	0/0		1.000	3/3		1.000	6/4.2		0.637
Recent head trauma	1/1		1.000	5/2		0.140	17/5.3		1.000
Epilepsy	0/0		1.000	1/6.7		0.361	3/20		0.037
Dementia	0/0		1.000	3/7.3		0.102	8/8.5		0.110
Arterial hypertension	1/1.5		1.000	8/3.2		0.733	18/6.3		0.118
Diabetes mellitus	0/0		1.000	2/2.4		1.000	9/8.7		0.070
Chronic kidney disease	0/0		1.000	1/3.6		1.000	6/9.5		0.115
Atrial fibrillation	0/0		1.000	2/2.9		1.000	10/8.3		0.081
Chronic heart disease	0/0		1.000	2/3		1.000	6/6.3		0.599
Cerebral vascular/transient ischemic accident	0/0		1.000	0/0		0.369	3/4.3		0.782
Operative technique (one burr hole)	1/1		1.000	5/2.2		0.015	13/4.9		0.446
Year of surgery	-		0.595			0.203	-		0.356
CSDH location (bilateral)	1/3.6		0.346	1/1.3		0.471	4/5.1		1.000
Prior AAT use	0/0		0.621	6/3.3		0.754	17/6.8		0.057
Perioperative AAT short stop and heparin bridging	NA		NA	0/0		1.000	1/25		0.193
No perioperative stop of AAT	0/0		1.000	0/0		1.000	0/0		1.000
At admission: mRS > 3	1/2.8		0.431	8/6.1		0.007	16/6.5		0.155
Preoperative symptoms and signs									
-Nausea	0/0		1.000	0/0		0.648	1/7.7		1.000
-Headache	1/1.1		1.000	1/0.8		0.105	4/4.1		0.618
-Dizziness	0/0		1.000	1/2		1.000	1/2.2		0.490
-Change in behavior	0/0		1.000	2/3.6		1.000	3/5.1		1.000
-Cognitive deterioration	1/2.6		1.000	5/4.4		0.307	8/5.4		1.000
-Gait disorder	1/1.8		1.000	3/1.9		0.350	8/3.6		0.162
-Seizure < 24 h	0/0		1.000	2/8		0.157	0/0		0.628
-Neurological deficits	1/1.4		1.000	6/2.8		1.000	11/4.2		0.327
-Altered state of consciousness	1/2.1		1.000	8/5.1		0.048	14/6.8		0.167
-GCS score ≤ 7	0/0		1.000	2/33.3		0.009	1/20		0.236
-Aphasia	0/0		1.000	4/3.6		0.733	11/8		0.092
-Dysarthria	0/0		1.000	1/3.7		1.000	3/5.6		1.000
-Hemiparesis	1/1.6		1.000	7/3.7		0.352	15/6.3		0.244
-Hemiplegia	0/0		1.000	1/33.3		0.084	0/0		1.000
-Sensitive deficit	0/0		1.000	0/0		0.403	1/2.3		0.494
Preoperative CT-based severity criteria									
-Hematoma thickness (mean ± SD in mm)	24.4 ± 2.3		0.556	19.9 ± 8.4		0.106	27.1 ± 9.5		0.768
-Midline shift > 10 mm	0/0		0.532	3/3.4		1.000	6/7.8		0.397
-Presence of septations	0/0		0.500	5/2.8		1.000	11/5.7		1.000
-Density (mixed)	0/0		0.186	7/3.5		0.499	15/6.2		0.604
Postoperative complications									
-Seizure	0/0		1.000	2/9.5		0.117	2/6.9		1.000
-Bleeding requiring re-operation	1/16.7		0.080	3/9.1		0.059	6/33.3		<0.001
-Pneumonia	0/0		1.000	1/12.5		0.211	4/13.8		0.054
-Pulmonary embolism	0/0		1.000	0/0		1.000	1/25		0.193
-Myocardial infarction	0/0		1.000	0/0		1.000	1/33.3		0.148
-Stroke	0/0		1.000	0/0		1.000	1/50		0.101
First recurrence	0/0		1.000	1/1.7		0.704	3/5.9		1.000
Second recurrence	0/0		1.000	1/9.1		0.279	2/50		0.014
Third recurrence	0/0		1.000	1/100		0.029	1/100		0.052
(**B**)									
**Multivariate Analysis**									
	**Age Groups (Years)**								
	**18–64** **(*n* = 147)**			**65–79** **(*n* = 347)**			**≥80** **(*n* = 385)**		
**Predictors**	**aOR**	**95% CI**	***p*-Value**	**aOR**	**95% CI**	***p*-Value**	**aOR**	**95% CI**	***p*-Value**
Age	NA	NA	0.151						
Charlson comorbidity index	3.3	0.8–13.2	0.092	NA	NA	0.173			
ASA status				NA	NA	0.433	NA	NA	0.255
Epilepsy							NA	NA	0.078
Diabetes mellitus							NA	NA	0.104
Atrial fibrillation							2.9	1.1–7.8	0.031
Prior AAT							NA	NA	0.257
Operative technique				NA	NA	0.056			
mRS at admission (mRS > 3)				9.6	1.1–83.6	0.041			
Preoperative altered state of consciousness				NA	NA	0.756			
Preoperative aphasia							NA	NA	0.074
Preoperative hemiplegia				NA	NA	0.486			
Preoperative GCS score ≤ 7				8.4	1.1–63.9	0.040			
Postoperative bleeding requiring re-operation	NA	NA	0.111	NA	NA	0.331	NA	NA	0.129
Postoperative pneumonia							15.4	4.8–49.8	<0.001
Second recurrence							NA	NA	0.060
Third recurrence				NA	NA	0.999	NA	NA	0.097

Abbreviations: AAT: antiplatelet and/or anticoagulation therapy, ASA status: Anesthesiologists Physical Status Classification System, CSDH: chronic subdural hematoma, CT: computed tomography, GCS: Glasgow coma scale, IQR: interquartile range, mRS: modified Rankin scale, SD: standard deviation, aOR: adjusted odds ratio, CI: confidence intervals, NA: not applicable.

**Table 3:** (**A**): Predictors of poor functional outcome at discharge. (**B**): Predictors of poor functional outcome at discharge.

(**A**)									
**Variables**	**Age Groups (Years)**								
	**18–64** **(*n* = 147)**			**65–79** **(*n* = 347)**			**≥80** **(*n* = 385)**		
**Univariate Analysis**									
	**Mean ± SD**		***p*-Value**	**Mean ± SD**		***p*-Value**	**Mean ± SD**		***p*-Value**
Age (mean ± SD)	58.5 ± 7.2		0.038	74.2 ± 4.3		0.012	85.3 ± 3.7		0.004
	**Median (IQR)**		***p*-value**	**Median (IQR)**		***p*-value**	**Median (IQR)**		***p*-value**
Charlson comorbidity index (median, IQR)	2 (2)		0.075	5 (3)		<0.001	5 (2)		<0.001
ASA status (median, IQR)	3 (0)		<0.001	3 (0)		<0.001	3 (0)		<0.001
	**n/%**		***p*-value**	**n/%**		***p*-value**	**n/%**		***p*-value**
Sex (female)	9/25.7		0.049	24/23.8		0.242	73/51		0.459
Recent head trauma	11/12.1		0.467	70/27.6		0.687	181/56.2		0.038
Epilepsy	4/36.4		0.052	9/60		0.009	13/86.7		0.015
Dementia	0/0		1.000	25/61		<0.001	72/76.6		<0.001
Arterial hypertension	9/13.4		0.818	72/28.7		0.792	161/56.5		0.081
Diabetes mellitus	4/25		0.248	35/42.7		0.001	65/63.1		0.029
Chronic kidney disease	1/50		0.266	14/50		0.010	41/65.1		0.054
Atrial fibrillation	0/0		0.634	27/38.6		0.038	67/55.8		0.659
Chronic heart disease	2/16.7		1.000	23/34.8		0.224	58/60.4		0.156
Cerebral vascular/transient ischemic accident	2/18.2		1.000	21/44.7		0.009	36/52.2		0.791
Operative technique (one burr hole)	11/11		0.211	70/30.2		0.110	135/51.3		0.243
Year of surgery	-		0.833	-		0.045	-		0.089
CSDH location (bilateral)	5/17.9		0.766	14/18.4		0.043	45/57		0.530
Prior antiplatelet/anticoagulant therapy	7/18.9		0.416	61/33.3		0.031	143/57.2		0.070
Perioperative AAT short stop and heparin bridging	NA		NA	4/80		0.024	3/75		6.27
No perioperative stop of AAT	0/0		1.000	1/12.5		0.450	6/85.7		0.129
At admission: mRS > 3	17/47.2		<0.001	90/68.7		<0.001	194/78.5		<0.001
Preoperative symptoms and signs									
-Nausea	3/20		0.696	7/33.3		0.620	6/46.2		0.779
-Headache	6/6.6		0.001	17/13.8		<0.001	43/44.3		0.034
-Dizziness	1/4		0.128	9/17.6		0.091	17/37		0.018
-Change in behavior	5/29.4		0.071	22/40		0.049	38/64.4		0.089
-Cognitive deterioration	9/23.1		0.106	47/41.2		<0.001	94/63.5		0.003
-Gait disorder	11/20		0.147	51/31.7		0.191	116/52.5		0.606
-Seizure < 24 h	3/23.1		0.400	12/48		0.035	10/71.4		0.275
-Neurological deficits	15/20.3		0.058	72/33.8		0.005	145/56		0.231
-Altered state of consciousness	16/33.3		<0.001	67/42.7		<0.001	135/65.9		<0.001
-GCS score ≤ 7	1/50		0.235	6/100		<0.001	5/100		0.064
-Aphasia	9/31		0.008	37/33		0.202	89/64.5		0.002
-Dysarthria	2/33.3		0.205	12/44.4		0.073	34/63		0.185
-Hemiparesis	14/22.2		0.030	64/34.2		0.008	130/54.9		0.601
-Hemiplegia	0/0		1.000	3/100		0.022	4/66.7		0.690
-Sensitive deficit	5/21.7		0.327	8/22.2		0.442	22/51.2		0.747
Preoperative CT-based severity criteria									
-Hematoma thickness (mean ± SD in mm)	23.1 ± 8.9		0.836	24.1 ± 9.8		0.220	28.1 ± 15		0.057
-Midline shift > 10 mm	8/16		0.616	30/33.7		0.337	44/57.1		0.794
-Presence of septations	9/14.5		1.000	56/31.8		0.377	108/55.7		0.912
-Density (mixed)	12/15.6		0.623	68/34.3		0.018	142/58.7		0.053
Postoperative complications									
-Seizure	5/45.5		0.010	14/66.7		<0.001	23/79.3		0.006
-Bleeding requiring re-operation	1/16.7		1.000	15/45.5		0.026	22/75.9		0.019
-Pneumonia	0/0		1.000	7/87.5		<0.001	15/83.3		0.013
-Pulmonary embolism	0/0		1.000	1/100		0.282	4/100		0.127
-Myocardial infarction	0/0		1.000	0/0		1.000	3/100		0.252
-Stroke	1/100		0.143	0/0		1.000	2/100		0.502
(**B**)									
**Multivariate Analysis**									
**Predictors**	**aOR**	**95% CI**	***p*-Value**	**aOR**	**95% CI**	***p*-Value**	**aOR**	**95% CI**	***p*-Value**
Age	NA	NA	0.314	NA	NA	0.771	NA	NA	0.105
Sex (female)	4.3	1.1–17.4	0.040						
Charlson comorbidity index	NA	NA	0.470	1.3	1–1.6	0.036	NA	NA	0.697
ASA status	10.6	1.8–63	0.010	NA	NA	0.194	2	1.6–3.5	0.014
Recent head trauma							NA	NA	0.358
Epilepsy	11.4	1.3–101.5	0.029	NA	NA	0.542	19.1	1.5–242.3	0.023
Dementia				4.5	1.3–15.2	0.016	3.3	1.5–7.3	0.003
Arterial hypertension							NA	NA	0.246
Diabetes mellitus				NA	NA	0.593	NA	NA	0.277
Chronic kidney disease				NA	NA	0.680	NA	NA	0.189
Atrial fibrillation				NA	NA	0.609			
Cerebral vascular/transient ischemic accident				NA	NA	0.782			
Year of surgery				NA	NA	0.948	NA	NA	0.689
OP technique (burr hole)									
CSDH location (bilateral)				0.3	0.1–0.8	0.016			
Prior antiplatelet/anticoagulant therapy				NA	NA	0.265	NA	NA	0.401
Perioperative AAT short stop and heparin bridging				NA	NA	0.999			
mRS at admission (mRS > 3)	49.5	8.3–293.6	<0.001	79.5	28.3–223	<0.001	47.8	20.1–113.6	<0.001
Preoperative symptoms and signs									
-Headache	NA	NA	0.214	NA	NA	0.287	NA	NA	0.668
-Dizziness				NA	NA	0.869	NA	NA	0.076
-Change in behavior	NA	NA	0.085	NA	NA	0.098	NA	NA	0.784
-Cognitive deterioration				NA	NA	0.891	NA	NA	0.475
-Seizure < 24 h				NA	NA	0.119			
-Neurological deficits	NA	NA	0.334	NA	NA	0.737			
-Altered state of consciousness	NA	NA	0.195	NA	NA	0.951	2	1.1–3.8	0.035
-GCS score ≤ 7				NA	NA	0.377	NA	NA	0.309
-Aphasia	NA	NA	0.927				NA	NA	0.090
-Dysarthria				NA	NA	0.484			
-Hemiparesis	NA	NA	0.792	NA	NA	0.660			
-Hemiplegia				NA	NA	0.441			
Hematoma thickness							NA	NA	0.711
CSDH CT-density (mixed)				NA	NA	0.807	NA	NA	0.158
Postoperative complications									
-Seizure	29.5	2.8–311.7	0.005	NA	NA	0.132	NA	NA	0.277
-Bleeding requiring re-operation				NA	NA	0.062	7.2	1.7–30.8	0.007
-Pneumonia				38.8	2.2–695.3	0.013	NA	NA	0.420

Abbreviations: AAT: antiplatelet and/or anticoagulation therapy, ASA status: Anesthesiologists Physical Status Classification System, CSDH: chronic subdural hematoma, CT: computed tomography, GCS: Glasgow coma scale, IQR: interquartile range, mRS: modified Rankin scale, SD: standard deviation, NA: not applicable.

**Table 4:** Bootstrapped logistic regression for prediction of functional outcome at discharge in younger adults.

	Logistic Regression				Bootstrapped Logistic Regression			
Predictors	B	aOR	95%CI	*p*-Value	Bias	SE	*p*-Value	Bca 95% CI
Age	0.044	NA	NA	0.314	2.435	12.846	0.427	−4–25.9
Sex (female)	1.600	4.3	1.1–17.4	0.040	30.941	153.816	0.047	−19.6–330.3
Charlson comorbidity index	0.192	NA	NA	0.470	7.378	50.768	0.425	−21.2–80.4
ASA status	2.233	10.6	1.8–63	0.010	56.365	206.594	0.002	1.1–414.7
Epilepsy	3.100	11.4	1.3–101.5	0.029	108.019	453.970	0.008	−3.2–955.2
mRS at admission (mRS > 3)	4.931	49.5	8.3–293.6	<0.001	131.308	537.116	0.001	4–971.5
Preoperative Headache	−0.307	NA	NA	0.214	6.239	150.200	0.536	−143.7–204.2
Preoperative change in behavior	2.173	NA	NA	0.085	68.720	278.639	0.019	−0.5–528.7
Preoperative neurological deficits	−2.061	NA	NA	0.334	−70.445	383.031	0.071	−633.8–46.7
Preoperative altered state of consciousness	0.974	NA	NA	0.195	14.840	143.402	0.299	−137–276.8
Preoperative aphasia	−0.536	NA	NA	0.927	−22.385	150.895	0.443	−307.6–86.3
Preoperative hemiparesis	0.528	NA	NA	0.792	32.000	173.635	0.386	−35.2–361.7
Postoperative seizure	3.311	29.5	2.8–311.7	0.005	73.073	276.964	0.002	0.5–614.1

Abbreviations: ASA status: Anesthesiologists Physical Status Classification System, mRS: modified Rankin scale, NA: not applicable.

## Data Availability

The data presented in this study are available on request from the corresponding author.
